# Supplementary material for: Explaining why increases in generic use outpace decreases in brand name medicine use in multisource markets and the role of regulation
Source: PLoS One. 2024 May 2;19(5):e0301716. doi: 10.1371/journal.pone.0301716 (PMC11065256; doi:10.1371/journal.pone.0301716)
Supplement: S1 File — (DOCX) [file pone.0301716.s001.docx]

Explaining why increases in generic use outpace decreases in brand name medicine use in multisource markets
and the role of regulation

Katharina Blankart and Sotiris Vandoros

*March 27, 2024*

## S1 File. Description of counterfactual manner of oaxaca-blinder decomposition approach

This allows us to analyze counterfactual situations based on the alternative market structure function by analyzing what would be the distribution of changes in prescription medicine use rates if generics would increase as if they belonged to the brand name market. The groups of brand name and generic medicines that we compare are mutually exclusive such that we can interpret $Y_{\mathrm{Aij}}$ and $Y_{\mathrm{Bij}}$ as two potential outcomes for physician $i$ and active ingredient $j$ for which some assumptions are needed. With regards to the structural form, we assume that a active ingredient $j$ prescribed by physician $i$ belonging to either group $A$ or $B$ may be prescribed according to market conditions $m_{A}$ and $m_{B}$ which are functions of the medicines’ observable ($X$) and unobservable ($\varepsilon$) characteristics

$Y_{Aij}=m_{A}(X_{ij},\varepsilon_{ij})$ and $Y_{Bij}=m_{B}(X_{ij},\varepsilon_{ij})$

where $\varepsilon_{i}$ has a conditional distribution $F_{\varepsilon|X}$ given X, and $g=A,B$. Using this setting, we can decompose the difference in the market structure function between $A$ and into differences between the market structure functions, differences in the distribution of observable market characteristics ($X$) and differences in the distribution of unobservable characteristics ($\varepsilon$). In parallel to the potential outcomes framework, we can define simple counterfactual treatments. The counterfactual market structure then corresponds for example for example generic active ingredient $j$ ($D_{B}=1)$, observed change in prescription medicine use rates in the generic market is $Y_{B|D_{B},i}=m_{B}(X_{i},\varepsilon_{i})$ while the counterfactual change in use rates of brand name medicines would be $Y_{A|D_{B},i}^{C}=m^{C}\left( X_{i},\varepsilon_{i} \right)=m_{A}(X_{i},\varepsilon_{i})$.
