# Supplementary material for: Explaining why increases in generic use outpace decreases in brand name medicine use in multisource markets and the role of regulation
Source: PLoS One. 2024 May 2;19(5):e0301716. doi: 10.1371/journal.pone.0301716 (PMC11065256; doi:10.1371/journal.pone.0301716)
Supplement: S1 Table — Note: Prescription data was obtained from the CEGEDIM MEDIMED panel, 2011–2014. (DOCX) [file pone.0301716.s002.docx]

Explaining why increases in generic use outpace decreases in brand name medicine use in multisource markets
and the role of regulation

Katharina Blankart and Sotiris Vandoros

*March 27, 2024*

# Supporting Information

S1 Table: Active ingredients of multisource markets included in Decomposition Analysis, prescription medicine use rates, 2011-2014

| *ATC code* | *Non-proprietary name* | *prescriptions* | *expenditure before taxes and markups, Euro* | *patients* | *change in cost holding quantity constant* | *change in cost holding prices constant* |
| --- | --- | --- | --- | --- | --- | --- |
| A02BC02 | pantoprazole | 3,255,941 | 47,134,539 | 3,179,139 | 227% | (127%) |
| A02BC05 | esomeprazole | 265,391 | 6,688,353 | 256,024 | 40% | 60% |
| A10BF01 | acarbose | 53,625 | 1,214,182 | 50,831 | (1%) | 101% |
| A10BG03 | pioglitazone | 11,301 | 1,685,651 | 10,881 | 18% | 82% |
| B01AC04 | clopidogrel | 341,353 | 25,478,565 | 332,906 | 2% | 98% |
| C07AB12 | nebivolol | 362,750 | 2,624,863 | 357,907 | 1% | 99% |
| C08CA13 | lercanidipine | 441,404 | 5,863,817 | 426,027 | (15%) | 115% |
| C09CA01 | losartan | 342,452 | 10,814,581 | 337,041 | 7% | 93% |
| C09CA03 | valsartan | 699,288 | 22,371,455 | 685,809 | 28% | 72% |
| C09CA04 | irbesartan | 124,793 | 6,866,398 | 121,870 | 12% | 88% |
| C09CA06 | candesartan | 726,425 | 30,852,052 | 713,257 | 7% | 93% |
| C10AA04 | fluvastatin | 124,796 | 1,465,999 | 122,478 | 9% | 91% |
| C10AA05 | atorvastatin | 303,368 | 8,600,720 | 298,140 | 16% | 84% |
| G04BD07 | tolterodine | 28,415 | 2,405,752 | 27,469 | (3%) | 103% |
| J01FA10 | azithromycin | 453,100 | 1,255,824 | 439,197 | 17% | 83% |
| J01MA12 | levofloxacin | 292,244 | 1,519,039 | 279,571 | 1% | 99% |
| J05AB04 | ribavirin | 3,683 | 2,044,333 | 3,042 | 16% | 84% |
| L02BB03 | bicalutamide | 15,935 | 3,714,475 | 15,613 | 34% | 66% |
| L02BG03 | anastrozole | 28,401 | 5,654,252 | 27,914 | 9% | 91% |
| L02BG04 | letrozole | 24,627 | 5,752,525 | 24,142 | 6% | 94% |
| L02BG06 | exemestane | 14,771 | 3,650,562 | 14,483 | 9% | 91% |
| L04AA13 | leflunomide | 20,837 | 5,030,217 | 20,105 | 30% | 70% |
| L04AD02 | tacrolimus | 12,655 | 4,560,111 | 10,238 | 122% | (22%) |
| N02AA03 | hydromorphone | 87,139 | 14,097,612 | 76,035 | 6% | 94% |
| N02AE01 | buprenorphine | 113,406 | 13,519,718 | 95,089 | 13% | 87% |
| N02CC03 | zolmitriptan | 11,316 | 231,535 | 10,061 | (492%) | 592% |
| N02CC04 | rizatriptan | 72,083 | 1,691,323 | 67,769 | 57% | 43% |
| N03AF02 | oxcarbazepine | 50,418 | 1,333,601 | 44,610 | 60% | 40% |
| N03AX11 | topiramate | 34,603 | 2,304,536 | 32,473 | 88% | 12% |
| N03AX14 | levetiracetam | 134,102 | 21,365,396 | 124,208 | (1%) | 101% |
| N04BC05 | pramipexole | 117,944 | 15,156,783 | 105,060 | (79%) | 179% |
| N04BC06 | cabergoline | 10,705 | 1,251,953 | 10,091 | 82% | 18% |
| N05AE04 | ziprasidone | 6,563 | 1,827,495 | 6,074 | 4% | 96% |
| N05AH04 | quetiapine | 184,625 | 19,413,258 | 171,113 | (10%) | 110% |
| N05AX08 | risperidone | 208,074 | 7,437,836 | 192,929 | 26% | 74% |
| N06AB10 | escitalopram | 79,646 | 7,365,009 | 76,683 | (92%) | 192% |
| N06AX16 | venlafaxine | 242,308 | 8,150,936 | 235,021 | 19% | 81% |
| N06DA02 | donepezil | 49,824 | 7,154,416 | 48,384 | 14% | 86% |
| N06DX01 | memantine | 71,370 | 11,644,148 | 69,470 | (20%) | 120% |
| R03DC03 | montelukast | 99,305 | 7,556,019 | 96,400 | (8%) | 108% |
| R06AE09 | levocetirizine | 66,417 | 1,526,097 | 63,090 | (25%) | 125% |
| S01EE01 | latanoprost | 74,161 | 3,290,837 | 71,872 | 15% | 85% |

Note: Prescription data was obtained from the CEGEDIM MEDIMED panel, 2011-2014.
