# Supplementary material for: Explaining why increases in generic use outpace decreases in brand name medicine use in multisource markets and the role of regulation
Source: PLoS One. 2024 May 2;19(5):e0301716. doi: 10.1371/journal.pone.0301716 (PMC11065256; doi:10.1371/journal.pone.0301716)
Supplement: S2 Table — Note: The table displays the estimates of the adoption model to use a medicine by segment in 2014 having the medicine not used in 2011. Standard errors are in parentheses. * p<0.05, ** p<0.01, *** p<0.001. (DOCX) [file pone.0301716.s003.docx]

Explaining why increases in generic use outpace decreases in brand name medicine use in multisource markets
and the role of regulation

Katharina Blankart and Sotiris Vandoros

*March 27, 2024*

# Supporting Information

S2 Table: First Stage Partial Regression Estimates of likelihood to Newly Adopt Brand Name or Generic Medicine

|  | Prescriptions | Prescriptions |
| --- | --- | --- |
|  | Brand name | Generic |
| adopter |  |  |
| # ATC classes prescribing | -0.00045** | 0.00016 |
|  | (0.00016) | (0.00021) |
| # patients in practice | 0.00021*** | 0.00015*** |
|  | (0.00001) | (0.00001) |
| Physician is female | 0.05845*** | -0.00604 |
|  | (0.01364) | (0.01843) |
| Physician age | 0.00304*** | -0.00273* |
|  | (0.00089) | (0.00119) |
| Physician works in group practice | 0.06208*** | 0.03017 |
|  | (0.01684) | (0.02220) |
| Physician is specialist | 0.13561*** | 0.03139 |
|  | (0.01533) | (0.02001) |
| Months since market entry | -0.03284*** | 0.16854*** |
|  | (0.00157) | (0.00244) |
| Mean | -2.4771 | 6.5153 |
| N | 53,823 | 50,269 |
| Adoptions | 36,980 | 15,761 |
| chi2 | 6,040 | 5,850 |
| Lambda | 9.18 | -21.87 |
| Note: The table displays the estimates of the adoption model to use a medicine by segment in 2014 having the medicine not used in 2011. Standard errors are in parentheses. | | |
| * p<0.05, ** p<0.01, *** p<0.001 |  |  |
