# Supplementary material for: Explaining why increases in generic use outpace decreases in brand name medicine use in multisource markets and the role of regulation
Source: PLoS One. 2024 May 2;19(5):e0301716. doi: 10.1371/journal.pone.0301716 (PMC11065256; doi:10.1371/journal.pone.0301716)
Supplement: S3 Table — Note: The table shows estimates of the net differential in medicine use by brand name and generic medicines at active ingredient and physician level in 45 markets, 2011–2014. Compared to the baseline specification, it is assumed that brand name prescription medicines are replaced by generic prescriptions over time after patent expiry such that the differential in prescription medicine use is defined as Y¯B−−Y¯A. The analysis is identical to the Oaxaca-Blinder decomposition as section 2.4. For example, if generic utilization increases by two prescriptions and brand name utilization decreases by two, the differential prescription medicine use is zero. In the original specification, the utilization differential is four. Prescription data was obtained from the CEGEDIM MEDIMED panel, 2011–2014.; * p<0.05, ** p<0.01, *** p<0.001. (DOCX) [file pone.0301716.s004.docx]

Explaining why increases in generic use outpace decreases in brand name medicine use in multisource markets
and the role of regulation

Katharina Blankart and Sotiris Vandoros

*March 27, 2024*

# Supporting Information

S3 Table: Decomposition of differential in use of brand name compared to generic medicine, net prescription medicine use

|  | Prescriptions | Prescriptions SHI | Prescriptions / patient | Pharmaceutical expenditure |
| --- | --- | --- | --- | --- |
| overall |  |  |  |  |
| Brand name | 2.56*** | 2.35*** | 0.16*** | 231.39*** |
|  | (0.04) | (0.04) | (0.00) | (4.55) |
| Generics | 6.94*** | 6.27*** | 0.38*** | 128.06*** |
|  | (0.09) | (0.09) | (0.00) | (2.57) |
| Differential in use rates, 2011-2014 | -4.39*** | -3.92*** | -0.22*** | 103.33*** |
|  | (0.10) | (0.10) | (0.00) | (5.23) |
| adjusted |  |  |  |  |
| Brand name | 2.56*** | 2.35*** | 0.16*** | 231.39*** |
|  | (0.04) | (0.04) | (0.00) | (4.55) |
| Generics | 10.20*** | 9.41*** | 0.33*** | 182.34*** |
|  | (0.30) | (0.28) | (0.01) | (8.85) |
| Differential in use rates, 2011-2014 | -7.64*** | -7.06*** | -0.17*** | 49.06*** |
|  | (0.30) | (0.28) | (0.01) | (9.96) |
| Segment effects | -6.08*** | -5.81*** | -0.52*** | -183.74*** |
|  | (0.31) | (0.29) | (0.01) | (9.21) |
| Market structure | -11.69*** | -10.63*** | -0.82*** | -441.78*** |
|  | (0.31) | (0.29) | (0.01) | (13.19) |
| Interaction - segment and market structure | 10.13*** | 9.38*** | 1.17*** | 674.58*** |
|  | (0.32) | (0.30) | (0.01) | (12.96) |
| N | 95,949 | 95,949 | 95,949 | 95,949 |
| Brand | 52,411 | 52,411 | 52,411 | 52,411 |
| Generics | 43,538 | 43,538 | 43,538 | 43,538 |

Note: The table shows estimates of the net differential in medicine use by brand name and generic medicines at active ingredient and physician level in 45 markets, 2011-2014. Compared to the baseline specification, it is assumed that brand name prescription medicines are replaced by generic prescriptions over time after patent expiry such that the differential in prescription medicine use is defined as ${(\bar{Y}}_{B}-{(-\bar{Y}}_{A}))$. The analysis is identical to the Oaxaca-Blinder decomposition as section 2.4. For example, if generic utilization increases by two prescriptions and brand name utilization decreases by two, the differential prescription medicine use is zero. In the original specification, the utilization differential is four. Prescription data was obtained from the CEGEDIM MEDIMED panel, 2011-2014.; * p<0.05, ** p<0.01, *** p<0.001
