# Supplementary material for: Explaining why increases in generic use outpace decreases in brand name medicine use in multisource markets and the role of regulation
Source: PLoS One. 2024 May 2;19(5):e0301716. doi: 10.1371/journal.pone.0301716 (PMC11065256; doi:10.1371/journal.pone.0301716)
Supplement: S4 Table — (DOCX) [file pone.0301716.s005.docx]

Explaining why increases in generic use outpace decreases in brand name medicine use in multisource markets
and the role of regulation

Katharina Blankart and Sotiris Vandoros

*March 27, 2024*

# Supporting Information

S4 Table: Decomposition of differential in prescription medicine use rates by Generics compared to Brand Name Medicines

|  | Prescriptions | Prescriptions: SHI | Prescriptions / patient | Pharmaceutical expenditure |
| --- | --- | --- | --- | --- |
| overall |  |  |  |  |
| Brand name | -2.56*** | -2.35*** | -0.16*** | -231.39*** |
|  | (0.04) | (0.04) | (0.00) | (4.55) |
| Generics | 6.94*** | 6.27*** | 0.38*** | 128.06*** |
|  | (0.09) | (0.09) | (0.00) | (2.57) |
| Differential in use rates, 2011-2014 | -9.50*** | -8.62*** | -0.55*** | -359.45*** |
|  | (0.10) | (0.10) | (0.00) | (5.23) |
| adjusted |  |  |  |  |
| Brand name | -2.56*** | -2.35*** | -0.16*** | -231.39*** |
|  | (0.04) | (0.04) | (0.00) | (4.55) |
| Generics | 10.20*** | 9.41*** | 0.33*** | 182.34*** |
|  | (0.30) | (0.28) | (0.01) | (8.85) |
| Differential in use rates, 2011-2014 | -12.75*** | -11.76*** | -0.50*** | -413.73*** |
|  | (0.30) | (0.28) | (0.01) | (9.96) |
| Segment effects | -4.05*** | -3.57*** | -0.65*** | -490.84*** |
|  | (0.08) | (0.07) | (0.01) | (8.72) |
| Market structure | -6.67*** | -5.95*** | 0.02*** | -229.98*** |
|  | (0.42) | (0.39) | (0.01) | (13.22) |
| Interaction - segment and market structure | -2.03*** | -2.24*** | 0.13*** | 307.09*** |
|  | (0.32) | (0.30) | (0.01) | (12.39) |
| N | 95,949 | 95,949 | 95,949 | 95,949 |
| Brand | 52,411 | 52,411 | 52,411 | 52,411 |
| Generics | 43,538 | 43,538 | 43,538 | 43,538 |
