# Supplementary material for: Explaining why increases in generic use outpace decreases in brand name medicine use in multisource markets and the role of regulation
Source: PLoS One. 2024 May 2;19(5):e0301716. doi: 10.1371/journal.pone.0301716 (PMC11065256; doi:10.1371/journal.pone.0301716)
Supplement: Attachment — Submitted filename: Revision Note to PLOS One.pdf [file pone.0301716.s006.pdf]

**Revision Note to PLOS One, manuscript no PONE-D-23-12926**

**Explaining why increases in generic use outpace decreases in brand name medicine use in multisource markets and the role of regulation**

We thank the editor and the two reviewers for taking time to review our manuscript and the comments raised. All of these added strength to our approach and interpretation of our results. We performed the suggested changes to the mansucript and are confident that the changes improved the manuscript. Our fundamental changes were as follows:

1. We added prescriptions written to the publicly insured as outcome and refinded the reference price classification to reflect the situation at the beginning of our observation period, that means 2011. This led to recalibration of our analyses and somwhat modified results.

2. In greater detail, we explained the specification of market structure and segment effects.

3. Several minor changes and corrections have been directly addressed in the manuscript.

4. We added discussion points as suggested by the editor and the reviewers.

We present replies to all comments on the following pages.

## Additional Editor Comments

Thank you for this very interesting study. The peer review comments appear interesting and constructive.

I have only one comment to add from my side:

The authors demonstrate that the increase in generic use is greater (for many molecules) than the decrease in use of the respective brand name medicine. Throughout the paper, including nearly all of the Discussion, the implicit argument given is that the most likely explanation is inappropriate overuse of the generic.

In lines 466-468, the authors do note that it is possible that this same observation would be seen if there was inappropriate UNDERuse of the brandname prior to generic entry. Or to put it in simple terms, that for some patients, the expensive medicine was not received even when it would have been clinically appropriate, and now with lower prices more patients are receiving it (appropriately).

But it would seem that this potential explanation - of UNDERuse of brandname prior to generic entry - is at least as possible as the explanation given priority by the authors - that there is inappropriate OVERuse of the generic.

Please add discussion of why the data demonstrates that one of these explanations is more likely than the other. Or, if it is impossible to say which is more likely based on available data, please highlight these two as equally possible explanations.

> *Authors' response: Thank you very much for this note. We agree that both directions are possible, and we do not know the gold standard here. We have added the suggested perspective of underuse that could originate from substitution from other medicine classes that we do not account for, besides medical need, to the discussion as follows:*

> *"The increase in generic use may stem from overuse, particularly in instances of over-prescribing. Alternatively, it could be the result of previous underuse when medical needs were not adequately addressed or when a shift in demand occurred, with patients substituting other medicine classes after the entry of generics."*

Reviewers' comments:

Reviewer's Responses to Questions
**Comments to the Author**

1. Is the manuscript technically sound, and do the data support the conclusions?

The manuscript must describe a technically sound piece of scientific research with data that supports the conclusions. Experiments must have been conducted rigorously, with appropriate controls, replication, and sample sizes. The conclusions must be drawn appropriately based on the data presented.
Reviewer #1: Partly
Reviewer #2: Partly

---

2. Has the statistical analysis been performed appropriately and rigorously?
Reviewer #1: Yes
Reviewer #2: I Don't Know

---

3. Have the authors made all data underlying the findings in their manuscript fully available?

The PLOS Data policy requires authors to make all data underlying the findings described in their manuscript fully available without restriction, with rare exception (please refer to the Data Availability Statement in the manuscript PDF file). The data should be provided as part of the manuscript or its supporting information, or deposited to a public repository. For example, in addition to summary statistics, the data points behind means, medians and variance measures should be available. If there are restrictions on publicly sharing data—e.g. participant privacy or use of data from a third party—those must be specified.
Reviewer #1: No
Reviewer #2: Yes

---

*Author's response: We now provide a detailed data availability and provenance statement that describes the data collected and processed and provides code for data processing and analysis via: https://osf.io/euqs9/?view_only=523df531cc8541db9ca2c7d7ed4b334e*

4. Is the manuscript presented in an intelligible fashion and written in standard English?

PLOS ONE does not copyedit accepted manuscripts, so the language in submitted articles must be clear, correct, and unambiguous. Any typographical or grammatical errors should be corrected at revision, so please note any specific errors here.
Reviewer #1: Yes
Reviewer #2: Yes
5. Review Comments to the Author

## Reviewer #1:

Summary

The paper analyses an important topic on effects of drug regulation in Germany and contains interesting results on market design. It uses adequate data and statistical analysis. Overall, the paper is not always easy to follow. Variables should be defined and described more clearly and more explanations and discussions seem useful.

Major Issues

Page 8, line 160: It appears that prices of drugs are not explicitly taken into account, but implicitly based on the induced expenditure. Is this correct? If data on expenditure come from prescriptions, you might not consider substitution which bias the real expenditure. Please explain in more detail.

> *Authors' response: Our outcome variables are prescription volumes and expenditure, not prices, as we chose to perform our analyses at the physician level. Physicians can influence volume and expenditure (through volume), but not prices. We do not consider substitution that has taken place in the pharmacy based on prescription data, such that effective expenditure may be lower. Our results show expenditure levels based on physician choices and their responses to prices. We clarify this in the manuscript by stating:*
>
> *"We focus on prescribed instead of dispensed medicines to capture physician responses to market structure and segment effects and the resulting changes in prescription volume and expenditure. In considering the physician level, we capture expenditure levels before any substitution takes place at the pharmacy and physician responses to medicine prices as listed in prescription software."*

Page 8. Line 164: Which price level is used to calculate the expenditure (a) manufacturer or pharmacy selling price, b) gross price or net price, c) if net price: what is deducted: legally defined rebates, patient co-payment; discounts from tenders are not deducted)? What do you mean with "before taxes"? Is this the price without value added tax?

> *Authors' response: To be more concise, we consider net prices as listed by manufacturers. These exclude taxes including the value added tax, discounts and rebates. We did not deduct co-payments by patients. We clarified this in the text.*

Page 8, line 164: Have prices changes over time have been taken into account?

> *Authors' response: We consider listed prices as reported per month such that any price change is considered. We added a respective note to the text.*

Chapter 3.3.: The terms "adoption bias" and "market segment" are not precisely defined. How are the variable defining adoption bias and market segments coded?

> *Authors' response: The adoption bias is calcuclated as follows:* $(\hat{\theta}_B\hat{\lambda}_B - \hat{\theta}_A\hat{\lambda}_A)$. *In the text, we made this more explicit by stating:*

*"The term $\left(\hat{\theta}_B\hat{\lambda}_B - \hat{\theta}_A\hat{\lambda}_A\right)$ corrects for adoption bias to net out the estimated differences in conditional means due to selectivity of adoption at physician level."*

*To avoid confusion, we reintroduced the notation for market segments again at the beginning of this section: "We denote brand name medicines as market segment A and generics as market segment B."*

Chapter 3.4. The term "market structure" also used in Table 2 , for instance, should be defined more precisely. Which variable define which kind of market structure? How are those coded?

*Authors' response: We revised section 3.4 to clarify which variables belong to market structure and how they are defined. We included a note to Table 2 to indicate the variables that belong to market structure in the footnote.*

Page 13, lines 264-265: You use the share of privately insured patients to control for this factor. However, privately insured are a different market with different regulation. IRP, automatic substitution and regulations by the physicians associations do not apply to privately insured persons. You might discuss whether controlling for privately insured persons is enough in your study. However, I would suggest to exclude privately insured patients from the analysis to reduce bias, because your research question is in IRP which does not apply to privately insured patients.

*Authors' response: Thank you for this suggestion. As additional outcome, we introduced the number of prescriptions written to publicly insured patients to demonstrate differences in medicine use between the total market and the market where reference pricing applies. The partial regression results for this additional outcome suggest that while exact values somewhat differ, the direction and size of the effects are very similar when we consider prescriptions written to the publicly insured only.*

*In addition, we control for the share of privately insured as physicians may adopt different practices by how much their practice is exposed to privately insured patients. This observation has been made for the United States. For Germany, there is published evidence regarding differences in waiting times between the publicly and privatly insured.*

Page 18, line 361: A central result is that after adoption bias adjustment, the number of prescriptions per patient seems to be reduced by reference pricing. Table 1 shows the availability of generics which provides a cheaper alternative reduced brands and rises generic as well as overall prescriptions per patients. Why does the further impact of IRP do not work into the same directions? This appears counterintuitive. Please explain this central result in more depth in the results and/or discussions section,

*Authors' response: In the results presented, we always control for adoption bias, but we vary whether in addition to reference pricing, we control for additional market struture variables. In the new version of the paper where we use a different classification of reference price status (see explanation in the comment on page 6 of this revision note), the direction of the effects is identical in the partial regressions, regardless of whether we add market structure variables.*

Table 4: How do you define and code the variables "market structure" and "segments effects"?

*Authors' response: There is no Table 4 included in the manuscript but we added respective notes to Table 3.*

Page 23, line 459: Why is the increase in prescription volume not necessarily substantiated by medical need? How is this based on the data and analysis? One can regularly observe that the volume of prescriptions increase after generic entry and start of price competition in contrast to the monopoly of the patented brand. Economic theory suggests that the monopolistic price of the branded drugs results in higher price and lower volumes compared with the generic market structure which reflects competition. Based on this, the monopoly market structure is likely inducing undersupply not reflecting the medical need.

*Authors' response: In this section, we are referring to the substantial segment effects that we identify using the decomposition approach. We clarified this passage by replacing "Our results" by "The substantial segment effects that this study identifies"*

Page 24, line 475: specify the different market structure here. Some readers might focus n the conclusions section only.

*Authors' response: For clarity, we split this sentence in two and highlighted what we refer to as market structure.*

Suppl. A6: You use the 2012 version to capture reference pricing. Why not 2011? What about drugs being assigned to a IRP group between period 1 and 2?

*Authors' response: Thank you for this suggestion. The year 2012 was the oldest for which we could obtain a structured dataset of IRP within the study period. Generally, reference pricing data is difficult to obtain for Germany. We checked whether there were any active ingredients for which reference prices were newly introduced in the course of 2011 and 2012. We found this the case to be for three active ingredients. In that case, we coded reference price status to "no reference price" to reflect the situation in 2011. The goal of our study was not to study dynamics in prices subject to intorduction of a reference price, but quantity changes when an active ingredient is under a reference price regimen or not. As we compare 2011 and 2014, we considered the beginning of 2011.*

*The use of this classification leads to somewhat different results in the partial regressions and to in the Oaxaca-Blinder decomposition. We updated all Tables and the results section accordingly. We considered the new results in the interpretation of our analysis. All in all, the results are somewhat more aligned between different variants of the partial regressions (including market structure yes or no).*

Minor Issues

The conclusion in the abstract is vague. In which way should access to medicines be managed?

*Authors' response: We revised the abstract to be more specific with respect to our results.*

Different types of referencing are used. Reference Albrecht el al. in the supplement is not number [50].

*Authors' response: Sorry for the mistake. We added a separate reference section to the electronic supplement.*

Page 1, line 11: Is this based on gross or net prices. Do the expenditure reflect manufacturer or pharmacy retail prices.

*Authors' response: The number reflects gross prices. We added the number for net prices as well from the original source.*

Page 1, line 23: Market structure is more complex, i.e. automatic substitution, tenders, influence of regulation by physicians' associations (KV). This might be stated here.

*Authors' response: We agree that there are additional elements of market structure. In the text, we clarified that we are considering market structure elements that vary at the physician level by stating:*

*"While there are additional regulations such as automatic substitution by pharmacies, tenders or influence of regulatoin by physicians' associations, we concentrate on elements of market structure that vary at active ingredient level to influence physician prescription."*

Page 3, line 51: How is the choice set restricted by internal reference pricing (IRP)? What is the proposed mechanism? Physicians may prescribe whatever they see appropriate in Germany. In most situation, manufacturers lower their prices such that patients do not have to pay a positive difference between prices and reference price.

*Authors' response: We agree that within each reference price group, differences in prices become small once the reference pricing mechanism has been introduced. Here, we take the view across difference medicines or medicine classes that may be subject to a reference price regulation or not. Given that physician behavior is often sticky, persistent or altruistic, physicians consider prescription of active ingredients subject to a reference price more carefully than unregulated medicines and stick to that practice. We added a note to the respective section by stating:*

*"Physicians may prescribe whatever they see appropriate, but may use medicines regulated under reference prices differently when patients may experience substitution or additional co-payments for these medicines when acting altruistically (10). In addition, physicians have been shown to be sticky to certain prescription patterns that may differ when a choice set is regulated compared to a free choice of options (11)."*

Page 5, lines 97: You might discuss potential incentives of regulation by physicans' associations. Quotas as used in Bavaria or "Richtgrößen" might induce incentives to prescribe more when generics become available. Generics can lower the average cost of prescriptions per patient such that there is more room to prescribe drugs for a patient, e.g. prescribing of two generic drugs to control blood pressure instead of using a branded combination or a single branded drug.

*Authors' response: We specified the respective passage by introducing references and referring to the mechanism of these policies by stating:*

*"In addition, prescription budgets (German: Richtgrößen) and minimum use rates of certain drug classes are monitored at physician level, but not access or volumes (24,25). These policies might induce incentives to prescribe more when generics become available."*

Page 6, line 125: The rebates do apply to all drugs, but are not equal. The rebate for generics can be lowered by lowering the price and no generic rebate applies for drugs priced 30% below the internal reference price.

*Authors' response: Thanks for this remark which we added to the text to be more precise.*

Page 6, line 136: Does this mean that your data reflect the specific drug prescribed by the physician and not the dispensed drug after automatic substitution?

*Authors' response: Yes, this is correct. Unlike data from sickness funds, our data reflect physician prescriptions only but does not contain dispensing information. This allows us to show changes in use rates by prescriptions induced by physicians. We agree that true pharmaceutical expenditure may be lower after substitution in the pharmacy. We added a respective note to the limitations section:*

*"In contrast to data from sickness funds, we derive pharmaceutical expenditure from prescription data. Accordingly, our estimates will be higher as we cannot account for automatic dispensing in the pharmacy to identify the cheapest option."*

Chapter 3.6.: Might be part of discussion after the results chapter.

*Authors' response: We moved the limitations section behind the discussion section and added a section of policy implications to the discussion.*

Table 1: Please explain the meaning of the variable "patients with indication".

*Authors' response: For clarity and consistency, in Table 1 and throughout all other resources we changed the label to patient structure as we describe the variable in the same way in the methods section.*

Table 2: put "brand" and "generic" between the columns to which these refer.

*Authors' response: We added "brand" and "generic" to each column to avoid convusin.*

Page 20, line 390; page 21, line 412/412: It seems that an "if" is missing in the senctence.

*Authors' response: We corrected the sentence accordingly.*

Page 20, line 392: What do you mean with "endowments"? Please define more precisely.

*Authors' response: Endowments is the term used in the classical Oaxaca-Blinder decomposition. We added a note referring to this in the text.*

## Reviewer #2:

This paper uses a data set on prescription data from Germany to examine the utilization patterns of brand and generic drugs. The study tackles a pertinent issue and offers noteworthy insights, thereby warranting its publication. Nevertheless, the paper's contribution and the discussion of its findings would benefit from being more prominent. In the following section, I elaborate on this issue and list several minor comments.

> *Authors' response: Thank you very much for reviewing our paper and the comments made.*

My main criticism is that the paper lacks a clear discussion of the results in relation to the existing literature. The authors state that their contribution lies in:
• Considering physicians' prescribing behavior.
• Considering the German market (previous studies focused on the USA).
The discussion only partially touches upon these points, and there is a lack of clear comparison with the existing literature. The authors should elaborate on this.

> *Authors' response: Thank you very much for this suggestion. We added two paragraphs to the discussion section that reflects our results in comparison to studies in these categories (p. 27).*

Minor comments:

The findings section in the abstract has the potential to mislead readers. It should be highlighted that while generics are effective in reducing expenditures, they come at the cost of increased net prescription rates (provide both delta prescriptions and delta expenditures).

> *Authors' response: Thank you for this suggestion. We revised the abstract accordingly.*

Bar over YA, YB seems not be introduced as notation.

> *Authors' response: We added that "$\overline{Y}_{A,B}$ are the average observed levels of our outcomes for groups A and B."*

P16, line 343: Where do the percentages come from?

> *Authors' response: This is the mean across all calculated proportions of changes as shown in S1 Table. In the manuscript, we make this explicit now.*

Table 1: are these all variables the authors use? Please clarify what belongs to market structure (only the number of manufacturers?) etc. additional descriptive statistics would be helpful, i.e. upper and lower bounds.

> *Authors' response: We edited Table 1 to only include variables that were used in the regression analyses and added upper and lower bounds. We dropped the baseline levels of these variables in 2011 to keep clarity.*

"Estimates of reference pricing status are larger when 364 we do not control for market structure" So the number of competitors lowers the prices? Please elaborate.

*Authors' response: No, this is not the statement that we can draw from this analysis. In our analysis, we capture whether a medicine was subject to reference pricing or not. We do not show how prices evolve based on competition. We clarify this in the text by stating:*

*"This means that market structure biases the impact of reference price status on medicine use."*

Line 389: The paragraph solely presents technical arguments. What are the implications of these findings? Why would the same market structure lead to a decrease of -5.45 prescriptions?

*Authors' response: We revised the entire paragraph to be more explicit about the interpretation of the results.*
