# Supplementary material for: Explaining why increases in generic use outpace decreases in brand name medicine use in multisource markets and the role of regulation
Source: PLoS One. 2024 May 2;19(5):e0301716. doi: 10.1371/journal.pone.0301716 (PMC11065256; doi:10.1371/journal.pone.0301716)
Supplement: Attachment — Submitted filename: Revision Note to PLOS One.docx [file pone.0301716.s007.docx]

**Revision Note to PLOS One, manuscript no PONE-D-23-12926**

**Explaining why increases in generic use outpace decreases in brand name medicine use in multisource markets and the role of regulation**

Again, we thank the editor and the two reviewers for taking time to review our manuscript and the comments raised. We addressed all comments and present replies on the following pages.

# PLOS ONE Journal Requirements:

Please review your reference list to ensure that it is complete and correct. If you have cited papers that have been retracted, please include the rationale for doing so in the manuscript text, or remove these references and replace them with relevant current references. Any changes to the reference list should be mentioned in the rebuttal letter that accompanies your revised manuscript. If you need to cite a retracted article, indicate the article's retracted status in the References list and also include a citation and full reference for the retraction notice.

*Authors’ reaction: All references were reviewed and are complete and correct.*

# Additional Editor Comments:

Thank you for these thorough revisions. A few requests remain from Reviewer #2, which I hope are relatively easy to address.

Comments to the Author

# 1. If the authors have adequately addressed your comments raised in a previous round of review and you feel that this manuscript is now acceptable for publication, you may indicate that here to bypass the "Comments to the Author" section, enter your conflict of interest statement in the "Confidential to Editor" section, and submit your "Accept" recommendation.

Reviewer #1:All comments have been addressed

Reviewer #2: (No Response)

-------------------------

# 2. Is the manuscript technically sound, and do the data support the conclusions?

The manuscript must describe a technically sound piece of scientific research with data that supports the conclusions. Experiments must have been conducted rigorously, with appropriate controls, replication, and sample sizes. The conclusions must be drawn appropriately based on the data presented.

Reviewer #1: Yes

Reviewer #2: Yes

-------------------------

# 3. Has the statistical analysis been performed appropriately and rigorously?

Reviewer #1: Yes

Reviewer #2: Yes

-------------------------

# 4. Have the authors made all data underlying the findings in their manuscript fully available?

The PLOS Data policy [2] requires authors to make all data underlying the findings described in their manuscript fully available without restriction, with rare exception (please refer to the Data Availability Statement in the manuscript PDF file). The data should be provided as part of the manuscript or its supporting information, or deposited to a public repository. For example, in addition to summary statistics, the data points behind means, medians and variance measures should be available. If there are restrictions on publicly sharing data--e.g. participant privacy or use of data from a third party--those must be specified.

Reviewer #1: Yes

Reviewer #2: Yes

-------------------------

# 5. Is the manuscript presented in an intelligible fashion and written in standard English?

PLOS ONE does not copyedit accepted manuscripts, so the language in submitted articles must be clear, correct, and unambiguous. Any typographical or grammatical errors should be corrected at revision, so please note any specific errors here.

Reviewer #1: Yes

Reviewer #2: Yes

-------------------------

# 6. Review Comments to the Author

Please use the space provided to explain your answers to the questions above. You may also include additional comments for the author, including concerns about dual publication, research ethics, or publication ethics. (Please upload your review as an attachment if it exceeds 20,000 characters)

## Reviewer #1:

All comments have been adequately adressed. The paper gives clear insight into dynamics of the pharmaceutical market when generics enter.

## Reviewer #2:

The authors have addressed most of my comments appropriately. The comparison with literature is, however, still too vague and must be improved before publication. See comment 1.

Comment 1: Comparison against literature It is positive to have an overview of the related literature now.

However, the description of the papers' contributions is rather vague and challenging to follow. For instance, I examined Stargardt, reference 52, in "Considering the impact of reference pricing, the previous studies have demonstrated increases in health care use outside prescriptions at the patient level (52) and identified price reductions (53). Stargardt investigates "patients with prescriptions for statins."

*Authors’ response: We revised the paragraph starting at line 514 to more explicitly compare our study to the contributions of the studies cited. We added that Stargardt (52) examines physician and hospital visits to be more precise.*

To what metric/variables in (52) do you refer in "health care use outside prescriptions"?

*Authors’ response: We added that Stargardt (52) examines physician and hospital visits to be more precise.*

Likewise, the usage rate has not been defined in the paper. This is a central topic in the paper and should be briefly defined to avoid any misinterpretation.

*Authors’ response: In the introduction and background section, we clarify that we consider prescription volume and expenditure to reflect use rates of medicines. We added respective passages to the text in lines 51 and 90.*

Comment 2: Table 3

The values in Table 3 have considerably changed. I assume this is due to the recalibration mentioned in the note to the editor. Please clarify.

*Authors’ response: Yes, that is correct. We added prescriptions written to the publicly insured as outcome and refined the reference price classification to reflect the situation at the beginning of our observation period, that means 2011. This led to recalibration of our analyses and somewhat modified results that resulted in different magnitudes in the effects, but no changes in the qualitative interpretations of our results. We do not take any action with respect to the manuscript.*

-------------------------

# 7. PLOS authors have the option to publish the peer review history of their article (what does this mean? [3]). If published, this will include your full peer review and any attached files.

If you choose "no", your identity will remain anonymous but your review may still be made public.

Do you want your identity to be public for this peer review? For information about this choice, including consent withdrawal, please see our Privacy Policy [4].

Reviewer #1: No

Reviewer #2: No

-------------------------
